# Non-Wettable Microporous Sheets Using Mixed Polyolefin Waste for Oil–Water Separation

**DOI:** 10.3390/polym15143072

**Published:** 2023-07-17

**Authors:** Junaid Saleem, Zubair Khalid Baig Moghal, Rana A. Shakoor, Adriaan S. Luyt, Gordon McKay

**Affiliations:** 1 Division of Sustainable Development, College of Science and Engineering, Hamad Bin Khalifa University, Qatar Foundation, Doha P.O. Box 2713, Qatar; gmckay@hbku.edu.qa; 2 Center for Advanced Materials, Qatar University, Doha P.O. Box 2713, Qatar; zubairkhalid009@gmail.com (Z.K.B.M.); shakoor@qu.edu.qa (R.A.S.); aluyt@qu.edu.qa (A.S.L.)

**Keywords:** polyolefins, non-wettable, plastic wastes, thin sheets, oil spill, polyethylene, polypropylene

## Abstract

Mixed polyolefin-based waste needs urgent attention to mitigate its negative impact on the environment. The separation of these plastics requires energy-intensive processes due to their similar densities. Additionally, these materials cannot be blended without compatibilizers, as they are inherently incompatible and immiscible. Herein, non-wettable microporous sheets from recycled polyethylene (PE) and polypropylene (PP) are presented. The methodology involves the application of phase separation and spin-casting techniques to obtain a bimodal porous structure, facilitating efficient oil–water separation. The resulting sheets have an immediate and equilibrium sorption uptake of 100 and 55 g/g, respectively, due to the presence of micro- and macro-pores, as revealed by SEM. Moreover, sheets possess enhanced crystallinity, as evidenced by XRD; hence, they retain their structure during sorption and desorption and are reusable with 98% efficiency. The anti-wetting properties of the sheets are enhanced by applying a silane coating, ensuring waterless sorption and a contact angle of 140°. These results highlight the importance of implementing sustainable solutions to recycle plastics and mitigate the oil spill problem.

## 1. Introduction

Plastic pollution has become a growing concern globally. The amount of recycled plastic in developed countries is estimated to be less than 20%., with most of it destined for energy recovery [1]. According to the Environmental Protection Agency, plastic waste in the US accounts for 12.2% of municipal waste [2,3]. Plastic waste contributes to microplastic ingestion [4,5,6] and toxic chemicals being released into the environment [5,6,7]. By 2040, it is predicted that the amount of greenhouse gases released during the creation, consumption, and disposal of traditional plastics made from fossil fuels will increase to account for 19% of the world’s carbon budget [8]. Therefore, unless proper recycling measures are taken, the risks associated with plastic waste’s environmental impacts will rise exponentially, due to waste amalgamation, in the coming decades [9,10].

Polyethylene (PE) and polypropylene (PP) are the two main types of plastics that make up 60% of all plastic waste [11,12,13,14]. They are often used for low-end, one-time applications and are commonly discarded as waste. Due to their similar densities and immiscibility, they are difficult to separate and blend [15,16,17,18,19,20,21]. If they are melted together, they tend to phase separate, forming distinct regions of each polymer, rather than forming a single, homogenous melt. This can result in a non-uniform material with varying properties, which may be undesirable. However, the compatibility issues can be resolved by preparing a dilute solution of PP and PE without using a compatibilizer. This method avoids conventional blending techniques, such as extruders and melt-blenders. By dissolving the polymer chains in a common solvent, it creates free space between the chains, making it possible to separate both polymers’ chains through the diffusion of the solvent molecules. The diluted solution prepared using this method can be used for a variety of applications, and one such application is the preparation of oil sorbent [12,22,23,24,25].

The use of polyethylene and polypropylene in the production of sorbents has been widely explored [26,27]. In this regard, Wei et al. [28] created a PP-based oil sorbent material and discovered that the oil sorbent’s effectiveness is correlated with the diameter of the PP fiber, oil property, and the porosity of the sorbent. Yuan et al. [26] developed nano-porous membranes, for which the wetting characteristics can be varied from super-hydrophobic to super-hydrophilic. In another study, super-hydrophobic mats made from electrospinning PP removed 99% of the water from fuel [29]. However, most of the polymeric sorbents reported in the literature had a limited oil uptake capacity. While commercial polymeric sorbent pads typically have a reasonable oil uptake capacity, they are not suitable for use in thin water-borne oil layers. As they have a minimum thickness of 10 mm, a portion of them hovers below the oil surface so they absorb unwanted water. Examples of such sorbent pads include Chemtex-BP-9W, 3M-156, and 3M-HP-255, which consist of stacked sheets to create a thick sorbent pad.

Hence, there is a need to prepare an oil sorbent that has a high oil uptake capacity. Our proposed technique addresses the following two problems simultaneously: (a) oil spill treatment, and (b) plastic waste. Among the available methods to create porous polymer sheets, the wet process yields uniform thickness and favorable physical properties [12,30]. We employed dissolution, thermally-induced phase separation, and spin-casting to prepare non-wettable microporous sheets with enhanced internal surface area. To assess the morphology and effectiveness, detailed characterizations via XPS, XRD, FTIR SEM, and tensile testing were carried out.

## 2. Experimental Section

### 2.1. Materials and Reagents

The p-xylene was procured from Sigma Aldrich and was used without further purification. PP and high-density polyethylene (HDPE) food containers were obtained from a local food restaurant, washed in soap solution and cut into small pieces for use as plastic waste sources. Diesel oil, WOQOOD 15w-40 engine oil, was purchased from a local petrol station. Paraffin oil was purchased from a local pharmacy store. N-hexane, 99.5% was purchased from Sigma and used as received for solvent washing of oil sorbents.

### 2.2. Method

#### 2.2.1. Thin Sheet Sorbent’s Preparation Process

**Figure 1:**
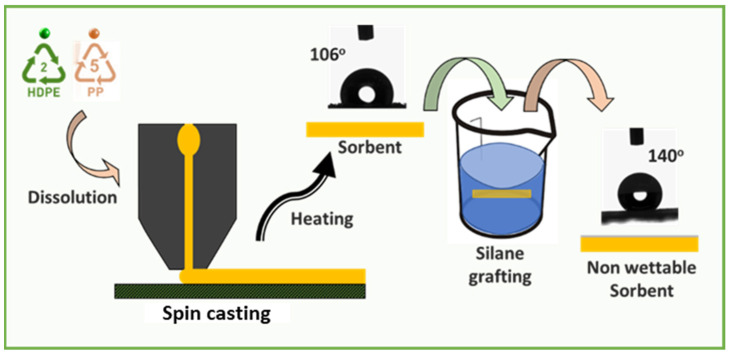
A method to fabricate hydrophobic and porous oil sorbent sheets.

In a conical flask, 1.5 g of HDPE and 1.5 g of PP were added. Then, 50 mL of p-xylene was added to the conical flask and heated under reflux conditions. A clear solution was obtained by heating the reaction mixture to 130 °C, but below the boiling point of the solvent used. The plastic waste polymer typically dissolved in 15 to 20 min. A reflux condenser was attached to a conical flask in order to minimize the loss of solvent. When the liquid had become evenly clear, it was then poured onto a pristine piece of glass that was placed on a specially designed spin coater chuck. To stop the solvent from evaporating and being exposed, the spin coater’s lid was sealed. To prevent solvent exposure to the surroundings, it is recommended to position the stirrer and spin coater adjacent to each other and to keep the entire apparatus within a fume hood. The spin-cast sheet was annealed in a hot air oven at 160 °C for 25 min to obtain an optimized microporous thin film sheet. The annealing can also be done on the Heidolph heating plate, but the duration required for annealing decreases to 15 s or less. Moreover, annealing of the thin sheet is a critical step and requires a detailed study before finalizing the optimum time and temperature as it leads to various porosities and strengths. After the spin-casting process, the thin sheet was detached from the glass substrate and further used for oil sorption studies, and the as-prepared thin film sheet was subjected to silanization to achieve a non-wettable surface. The details of the methodology can be found elsewhere [31,32,33]. Figure 1 represents the preparation of thin sheets.

#### 2.2.2. Oil Sorption Testing

A 25 cm^2^ as-prepared sorbent sheet was weighed and investigated for oil sorption studies. It was immersed in different types of sorbates (organic solvents and oils) for various durations to calculate saturation uptake. Then, the sorbent was taken out and oil uptake capacity was measured in the following two ways: (a) the sorbent was weighed right after being taken out from the oil bath, termed here as immediate uptake and (b) the sorbent was allowed to drain to remove loosely attached oil molecules, termed here as equilibrium uptake. The equilibrium values were obtained after 5 min of dripping as no more dripping was observed after 5 min. The oil uptake capacity was calculated as a mass of sorbate taken up by a unit mass of dry sorbent. The tests were carried out five times and an average value was reported in the results.

#### 2.2.3. Recyclability Test

The recyclability of the sorbent shows the durability and reusability for subsequent cycles. The reusability of the sorbent sheet was examined using the following two techniques: (a) mechanical squeezing, whereby the sorbent was squeezed mechanically to remove the oil from the sorbent sheet after oil sorption, and (b) solvent washing, whereby the sorbent was initially mechanically squeezed and was then placed in a hexane bath to remove the oil present in the microporous structure. Then, the same films were reused for oil sorption in subsequent cycles.

#### 2.2.4. Silane Grafting

The hydrophobicity of the sorbent was improved by grafting a silane layer on the surface of the oil sorbent film [34]. Initially, 100 µL of HDTMS and 150 µL of MTMS, 20 mL of water and 10 mL of ethyl alcohol were stirred in a beaker and the as-prepared thin sheet was dipped in it for 6 h to graft the silane layer onto its surface. Next, the film was removed and heated to 120 °C for 30 min to achieve a non-wettable superhydrophobic sorbent. The idea of silane grafting is to repel the water and absorb only the oil from the oil–water surface, resulting in selective and efficient oil sorption.

## 3. Results and Discussion

### 3.1. Composition Analysis of the HDPE–PP Sorbent through X-ray Diffraction

**Figure 2:**
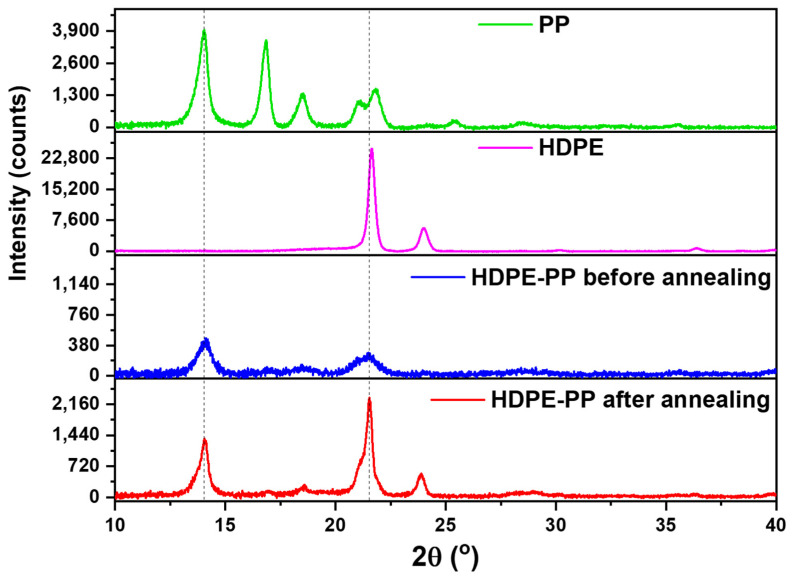
XRD spectra of HDPE-PP before and after annealing.

The XRD spectra of the HDPE–PP sorbent before and after annealing are shown in Figure 2. It was observed that the HDPE thin sheet sorbent before annealing showed a more amorphous nature, as compared with PP. The peaks at 13° and 22° are characteristic peaks of PP and HDPE, respectively [22]. Initially, the peaks before annealing were less intense and showed broader peaks compared to the peaks after annealing. In the annealed thin sheets, the characteristic peaks of PP and HDPE were enhanced significantly. This significant enhancement in peak intensity is attributed to the increase in crystallinity. The increase in crystallinity is due to the realignment and close packing of the polymer chains, through which the chains are compacted, resulting in increased crystallinity. The crystallinity before and after the annealing process was calculated through the area under the curve, and the calculated values are shown in Table 1. An increase of 24.9% in crystallinity is attributed to the rearrangement and compact alignment of polymer chains.

### 3.2. Morphological Analysis of PP, HDPE, and HDPE-PP through SEM

**Figure 3:**
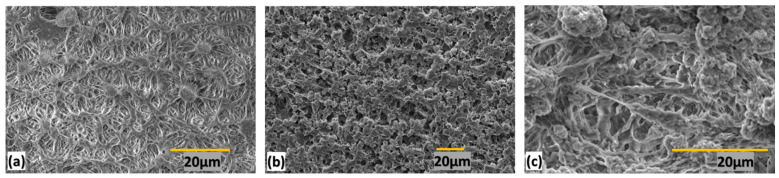
SEM images showing a porous network structure of (**a**) PP (**b**) HDPE (**c**) HDPE–PP.

The SEM images of PP, HDPE, and HDPE–PP, after annealing, are presented in Figure 3. The plastic PP showed a web-like network that was connected intermittently through polymer bridges, as shown in Figure 3a. Concurrently, the plastic HDPE appeared to have a flake-like or petal-like structure, as shown in Figure 3b. These petals were interconnected through intermolecular polymeric forces. Medianly, the HDPE–PP blend demonstrated a mesh-like structure, a hybrid morphology of both web-like and petal-like structures, as shown in Figure 3c. These intermolecular forces were the dispersion forces that created the interconnected structure through which the tensile strength was enhanced. As discussed in the X-ray diffractions section, the increase in crystallinity was due to the close and dense packing of the polymer chains, which was attributed to the enhancement in intermolecular forces, resulting in an interconnected morphological structure.

### 3.3. Functional Group Analysis of HDPE–PP before Annealing and after Annealing through FTIR

**Figure 4:**
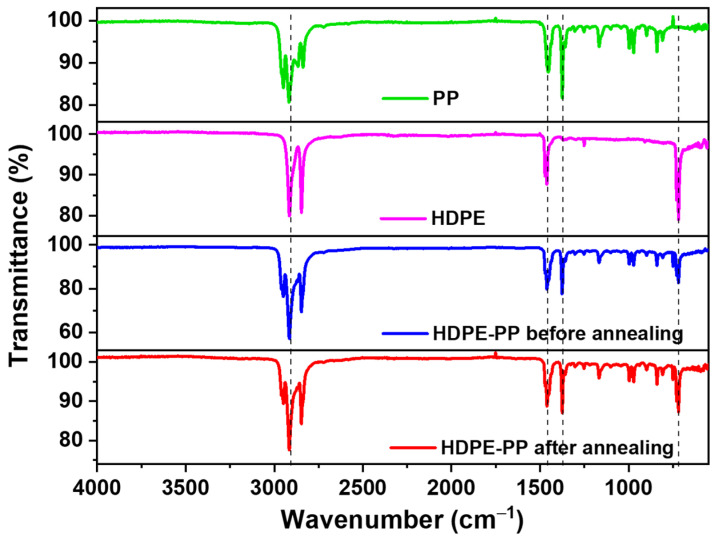
FTIR spectra of HDPE–PP before and after annealing.

The FTIR spectra of the HDPE–PP, before annealing and after annealing, were compared with PP and HDPE, as shown in Figure 4. It was observed from the PP scan that the C–H peaks were in the region of 2950–2850 cm^−1^, and the C–H bending peaks were observed at 1450 cm^−1^ and 1370 cm^−1^. Moreover, the branched methyl -CH_3_ stretching was found only in PP in the region of 2950 cm^−1^. Similarly, in the HDPE scan, the C–H stretching peaks were observed in the regions of 2930 cm^−1^ and 2850 cm^−1^, respectively, and the C–H bending peaks were observed at 1454 cm^−1^. Unlike the case in PP, HDPE represented intense C–H peaks in the region 730 cm^−1^. The characteristic peaks of PP and HDPE in HDPE–PP presented the CH stretching and branched free methyl -CH_3_ peaks in the region between 2950–2850 cm^−1^. The C–H bending peaks and C–H rocking peaks were observed in the regions of 1452 cm^−1^ and 730 cm^−1^, respectively. However, the transmittance (T%) in the before-annealing HDPE–PP was very low compared to the after-annealing blend. This low transmittance and more absorbance in the before-annealed thin film was attributed to the more amorphous character of the polymer blend. A more amorphous character results in a distorted arrangement of the polymer chains, resulting in more diffractions and more scattering, and more absorption of energy. On the other hand, the after-annealed thin film showed more crystallinity, which was due to the compact and proper arrangement of the polymer chains, resulting in fine alignment and more transmission [35]. Thus, higher crystallinity in the after-annealing blend suggested the dense and compact configuration of polymer chains, leading to an increase in strength.

### 3.4. XPS Analysis of HDPE–PP before and after Annealing

**Figure 5:**
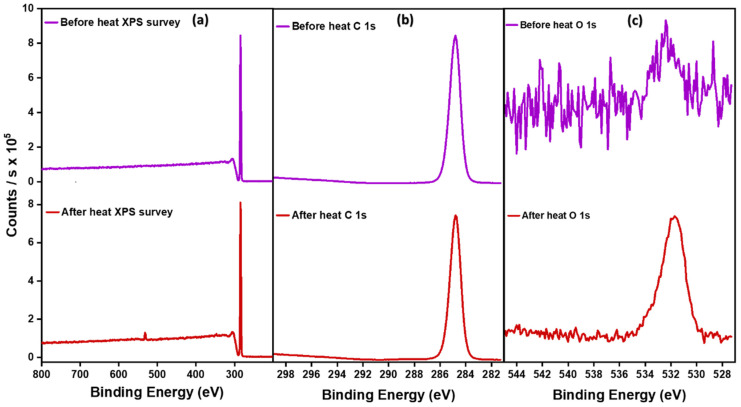
(**a**) XPS survey, (**b**) C1s, (**c**) O1s spectra of HDPE–PP before and after annealing.

The XPS spectra of the HDPE–PP before and after annealing are shown in Figure 5. A peak at 292 eV, which was due to π–π* interactions of aromatic conjugated bonds inferred the presence of xylene [36]. No peak was observed at 292 eV in the before and after annealing samples, confirming solvent removal during the spin-casting process. The C 1s spectra represented the presence of carbon species like C-C, C-C-O, etc at 284 eV. Moreover, the existence of a very small amount of oxygen in the after-annealing sample was identified at 532 eV. We further explored O 1s spectra to identify the type of oxygen species, which was identified to be the C-C-O or C-O-C ether functional group. The small quantity of oxygen detected was attributed to the partial oxidation of the sorbent that took place during annealing.

### 3.5. Determination of Mechanical Properties of HDPE–PP Sheets

Oil sorbent sheets depend on their tensile strengths. When thin sheet sorbents were prepared at room temperature, they showed 78% porosity with no mechanical strength. However, these thin sheets could not be used for practical purposes, because of their insufficient tensile strength. When attempts were made to remove the thin films from the substrate, chipping of the polymer occurred and intact film was not obtained. Later, we tried to enhance the tensile strength by annealing the thin sheets, which eventually compacted the thickness of the sheets. Annealing caused shrinkage in polymer chains, reducing the porosity to 73%. This compacted the sheet thickness, yet the desired strength could not be achieved. After that, the annealing duration was increased by another 10 min, which led to relatively more shrinkage of polymer chains and decreased porosity and thickness. However, we noticed a discernible improvement in the tensile strength of the sheets at that time. The sheets were reinforced by increasing the annealing time to 15 min. The optimum values of tensile strength were achieved with an annealing period of 20 min. Beyond that, porosity and thickness were significantly reduced, as shown in Table 2. The optimized values were obtained with 33% porosity, 7 μm thickness, and 8 MPa tensile strength, which were sufficient for real-time sorbent applications. These thin films are highly efficient when the oil-water separation is in very low quantities, and also when the oil is in very thin layers. For these thin layers of oil, thick pads are insufficient, because most of the pad area is submerged in the water, making much less contact with the oil and, hence, inefficient in oil–water separation. Thus, a very thin film sorbent makes a perfect solution for the separation of thin oil layers from the water bodies.

**Figure 6:**
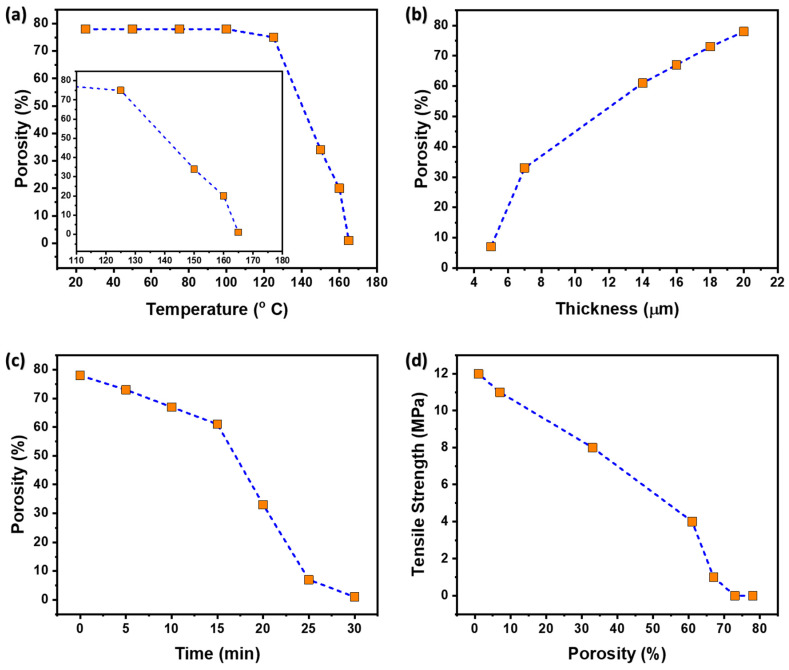
Factors that affect porosity (**a**) temperature vs. porosity (**b**) thickness vs. porosity (**c**) time of annealing (**d**) porosity vs. tensile strength.

Figure 6a–d shows the effects of annealing temperature on porosity, thickness on porosity, annealing time on porosity, and porosity on strength, respectively. High porosity could be achieved at temperatures ranging from 20–120 °C; however, as the temperature further increased, the sorbent porosity decreased, because of the shrinking of polymer chains, as shown in Figure 6a. However, with an increase in thickness, the pore size and the pore size distribution increased, resulting in increased porosity, as shown in Figure 6b. In contrast, upon annealing, the polymer became soft and the pores started shrinking, resulting in a decrease in thickness. Thus, when the polymer is annealed porosity decreased. The time of annealing also plays a major role in maintaining the porosity. When the controlled heating increased, the polymer molecules absorbed more and more energy, thus becoming soft and compact, and, thereby, the pore size and pore size distribution decreased, resulting in a decrease in porosity, as shown in Figure 6c. Figure 6d exhibits the relationship between porosity and tensile strength. Higher porosity tends to have low tensile strength because of the weakened internal structure of the material. The weak internal structure is a result of minimal intermolecular forces and loosely connected polymeric bridges. These bridges are strengthened when annealed, due to an increase in dispersion forces or the van der Waal forces; thus, resulting in more tensile strength and decreased porosity.

### 3.6. Contact Angle Measurements

**Figure 7:**
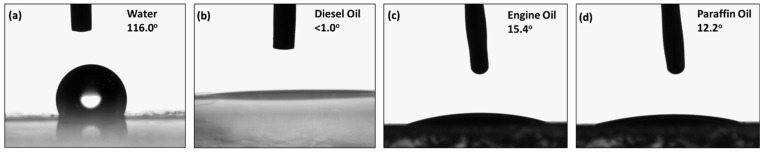
Contact angle of (**a**) water (**b**) diesel oil (**c**) engine oil, and (**d**) paraffin oil on the HDPE–PP thin film sorbent surface.

The HDPE–PP thin sheet sorbent showed a contact angle of 116°, which revealed the surface as hydrophobic, but not superhydrophobic. However, the oil tests confirmed the super oleophilic nature of the sheet. When tested with diesel oil, the contact angle was found to be <1.0°; hence, it was difficult to capture the contact angle. As soon as the droplet came in contact with the thin sheet surface, the diesel oil spread over the thin sheet in a fraction of a second without creating any meniscus. This flattening suggested that these sheets were super oleophilic. Further, the contact angle was also measured with paraffin oil and engine oil, for which we found the contact angles of 12.2° and 15.4°, respectively, and the angles decreased with time. This suggested that these sheets were microporous and could absorb oil. Moreover, the affinity of this thin film sheet towards oil was more than that of water. Thus, the sheets could be used in oil–water separation and oil sorption studies. However, when the film came into contact with an oil and water body, wherein the oil was in very low quantities, it absorbed 97 ± 2% oil and some water also adhered to the film, due to oxygen species. The contact angle images are shown in Figure 7a–d.

### 3.7. Process of Oil Sorption

**Figure 8:**
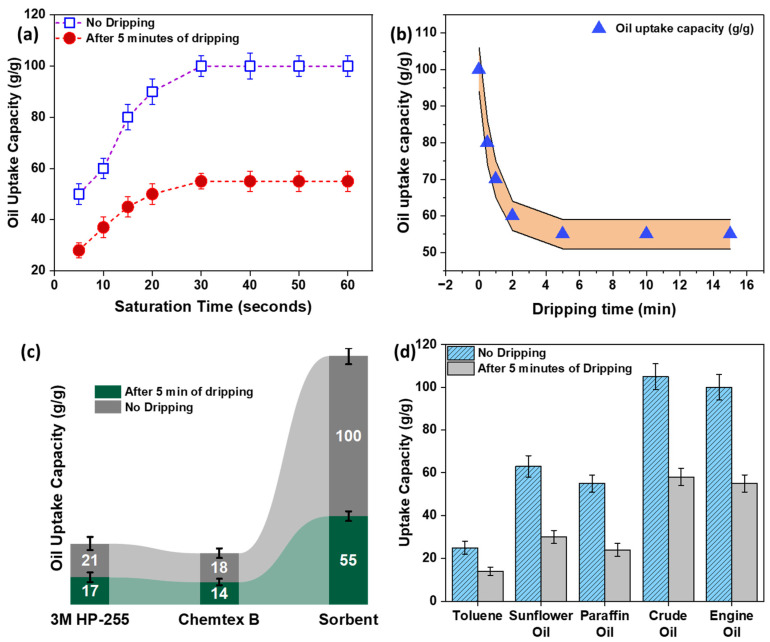
The oil sorption studies of HDPE–PP thin sheet sorbent (**a**) saturation kinetics (**b**) dripping kinetics (**c**) comparison using commercial sorbents (**d**) uptake capacity using different oils.

Saturation kinetics refers to the time taken by a thin sheet sorbent to reach a saturation point, whereafter, the oil cannot be further absorbed. In short, it is the maximum capacity of the oil absorbed by a thin sheet with respect to time. In other words, the total amount of oil absorbed by a sorbent in a specific time. The saturation time gives us an understanding of how fast a sorbent can absorb oil. The larger the surface area, the faster the absorption. In this study, as the sorbent was in the form of a thin sheet, the surface area was high, compared to an aerogel, where the contact surface area is limited. In thin sheet form, the contact surface area between the sorbent and oil is very high, thus reaching saturation in a short time. Whereas in the aerogel the contact surface area between the oil and the aerogel is low, and the oil is absorbed through capillary action; thus, the sorption time is high. The as-prepared thin sheet sorbent reached its saturation value within 30 s of sorption. The saturation capacity at different times is shown in Figure 8a.

Dripping kinetics refers to the amount of oil dripping from a thin sheet sorbent over time. A thin sheet of sorbent was placed in oil and was allowed to absorb oil till it reached saturation. After it reached equilibrium, the thin sheet of sorbent was taken out, and loosely connected oil was allowed to drain from it. Initially, the oil drips more, and, as time passes, the dripping decreases, and then, after some time, some oil is retained by the sorbent sheet, and the oil does not drip. This is termed the retention capacity of the thin sheet sorbent. The as-prepared thin sheet sorbent reached its equilibrium uptake value after 5 min of dripping, i.e., after 5 min there was no more dripping of oil from the sorbent. Similarly, the larger the contact surface area, the higher the binding affinity of the sorbent to the oil, resulting in more uptake capacity. The retention capacity, or dripping kinetics, at different times is shown in Figure 8b. Engine oil (density 0.89 g/cm^3^) was used to carry out the saturation and dripping studies.

A comparison of the as-prepared oil sorbent sheet with commercial sorbents CS1 and CS2, namely 3M255 and ChemtexBP9W, is reported in Figure 8c. The equilibrium oil uptake value of our as-prepared thin sheet sorbent was 55 g/g, which was much higher than that of the commercial sorbent pads. The uptake capacity for the HDPE–PP thin sheet sorbent was investigated using different oils and liquids, and the values are shown in Figure 8d. The results showed that the sorbent thin film was effective in absorbing diverse oils from much less viscous to viscous oils, edible to mineral, crude to automotive oils, etc. The more viscous the oil, the more the adhesion to the sorbent. The as-prepared sorbent followed an uptake mechanism which was a combination of the following: adhesion between oil and sorbent molecules, such that higher viscous oil tended to adhere more at the surface; cohesion between oil molecules trapped inside the pores and on the surface, resulting in a capillary action; higher surface area to thickness ratio, providing maximum available sites for oil sorption. The uptake capacity was measured soon after saturation was achieved and dripping was allowed for 5 min after reaching equilibrium.

### 3.8. Recyclability

**Figure 9:**
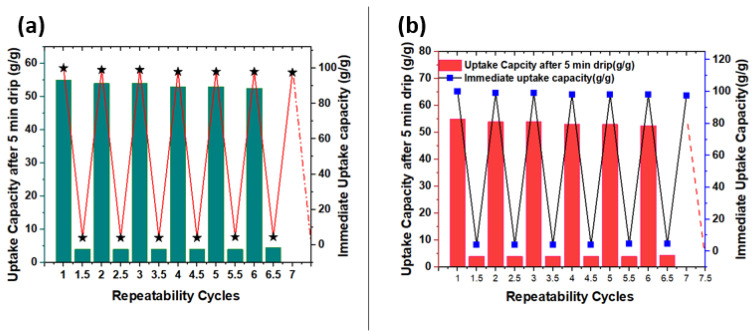
Recyclability of HDPE–PP thin film sorbent through (**a**) solvent washing method, and (**b**) mechanical squeezing using engine oil.

The recyclability of the as-prepared thin sheet sorbent, using mechanical squeezing and hexane washing, is presented in Figure 9. The sorbent was dipped in engine oil till saturation was achieved. The sheet was then removed from the oil and weighed immediately. The oil uptake capacity immediately after the sheet was taken from the oil surface was found to be 100 g/g, and the oil uptake capacity after reaching equilibrium was found to be 55 g/g. Then, the thin sheet of sorbent was mechanically squeezed to remove oil from it and weighed again. Mechanical squeezing resulted in 96% oil recovery as some oil (4 g/g) was retained inside the pores of the sorbent sheet; whereas hexane washing led to 100% oil recovery. The repeatability was carried out for six more cycles and could be continued for more cycles. This robust characteristic of the thin film sorbent suggested that the sorbent could be used multiple times without losing its affinity and efficiency.

### 3.9. Contact Angle, Oil–Water Separation Study of Silane-Grafted Non-Wettable Sorbent

**Figure 10:**
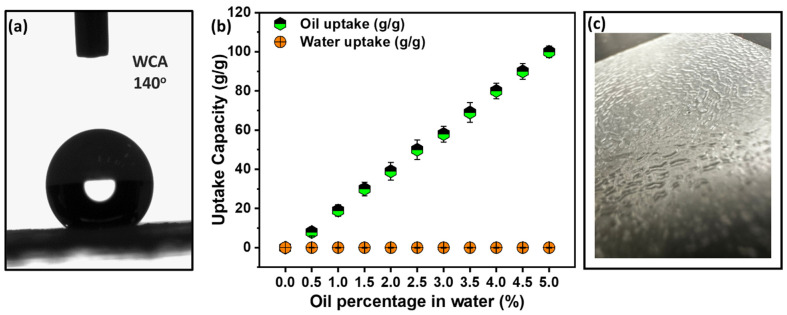
(**a**) The water contact angle on the non-wettable sorbent (silanized HDPE–PP thin film sorbent), (**b**) oil–water separation study showing highly effective oil only absorption characteristics of HDPE–PP silanized non-wettable thin film sorbent, (**c**) visual image of silane-coated non-wettable sorbent.

To further increase the oil sorption and water repulsion, the thin film sorbent was subjected to silane grafting, a well-known superhydrophobic agent. After the thin film sorbent was silanized, the non-wettable sorbent was subjected to contact angle measurements. The contact angle results revealed a significant improvement in hydrophobicity. The contact angle with water was found to be 140°, indicating a superhydrophobic nature that repelled water and was expected to solely absorb oil, as shown in Figure 10a. To demonstrate the sorbent’s selective oil uptake, an oil–water separation study was conducted, and is presented in Figure 10b. The non-wettable sorbent was immersed in an oil–water mixture ranging from 0.5–5% (*w*/*w*) oil in water, and it was observed that the sorbent absorbed only oil, with no water uptake. This experiment demonstrated the sorbent’s high oil selectivity over water, making it suitable for oil recovery in oil spill scenarios.

### 3.10. Oil Sorption in Previous Literature

A variety of plastic waste was used for the oil sorption, and some of the relevant reported literature is shown in Table 3.

## 4. Conclusions

Non-wettable microporous sheets using isolated HDPE and PP were prepared. These sheets have an enhanced internal surface area, due to the presence of micropores. Annealing ensures that the sheets have sufficient strength and can be used as free-standing structures. The sheets reached saturation in 30 s. The uptake capacity at the equilibrium point was found to be 55 g/g, though it varied with the type of oil. Further, the as-prepared sheets are recyclable, and more than 98% of oil can be easily squeezed out by means of mechanical squeezing.

This study targets reduction of the environmental impact of mixed polyolefin-based waste and addresses the challenges associated with oil spill remediation by valorizing waste into oil sorbent. Lastly, it expands the possibilities for upcycling plastic waste, which could be advantageous for managing waste during the shift towards a circular economy.

## Figures and Tables

**Table 1:** The % Crystallinity calculations using XRD data.

Polymer	% Crystallinity before Annealing	% Crystallinity after Annealing	% Change in Crystallinity
HDPE–PP	35.5	60.4	24.9

**Table 2:** Porosity and Tensile Strength for HDPE–PP sheets.

Sr. No.	Porosity (%)	Annealing Temperature (°C)	Annealing Time (min)	Thickness (μm)	Tensile Strength (MPa)
1	78	25	0	20	ND *
2	73	160	5	18	ND
3	67	160	10	16	1
4	61	160	15	14	4
5	33	160	20	7	8
6	7	160	25	5	11
7	<1	165	5	5	12

* ND = not determined.

**Table 3:** Reported oil sorbents from various plastic wastes.

S.N.	Type of Plastic	Oil Uptake Capacity (g/g)	Type of Oil Used	Reference
1	PET	39.3	Chloroform	[37]
2	Recycled PET	40	Crude oil	[38]
3	Unsaturated Polyester Resins	18.3	Carbon tetrachloride	[39]
4	PP aerogel	6	Silicone oil	[40]
5	PE–PP sorbent	55	Engine oil	This work

## Data Availability

Not applicable.

## References

[1] Jehanno C., Alty J.W., Roosen M., De Meester S., Dove A.P., Chen E.Y.-X., Leibfarth F.A., Sardon H. (2022). Critical advances and future opportunities in upcycling commodity polymers. Nature.

[2] Ragaert K., Delva L., Van Geem K. (2017). Mechanical and chemical recycling of solid plastic waste. Waste Manag..

[3] (2021). Recycle Coach, 7+ Revealing Plastic Waste Statistics, Resources. https://recyclecoach.com/resources/7-revealing-plastic-waste-statistics-2021/#:~:text=Globally%20to%20date%2C%20there%20is,increased%20200%2Dfold%20by%202015.

[4] Rotjan R.D., Sharp K.H., Gauthier A.E., Yelton R., Lopez E.M.B., Carilli J., Kagan J.C., Urban-Rich J. (2019). Patterns, dynamics and consequences of microplastic ingestion by the temperate coral, Astrangia poculata. Proc. R. Soc. B Boil. Sci..

[5] Parashar N., Hait S. (2022). Plastic Waste Management: Current Overview and Future Prospects. Environ. Degrad. Chall. Strateg. Mitig..

[6] Jiang J., Shi K., Zhang X., Yu K., Zhang H., He J., Ju Y., Liu J. (2022). From plastic waste to wealth using chemical recycling: A review. J. Environ. Chem. Eng..

[7] Kataria N., Bhushan D., Gupta R., Rajendran S., Teo M.Y.M., Khoo K.S. (2022). Current progress in treatment technologies for plastic waste (bisphenol A) in aquatic environment: Occurrence, toxicity and remediation mechanisms. Environ. Pollut..

[8] UN Environment Programme (2022). Our Planet is Choking on Plastic.

[9] Xayachak T., Haque N., Parthasarathy R., King S., Emami N., Lau D., Pramanik B.K. (2022). Pyrolysis for plastic waste management: An engineering perspective. J. Environ. Chem. Eng..

[10] Zhang Y., Jiang H., Bian K., Wang H., Wang C. (2021). A critical review of control and removal strategies for microplastics from aquatic environments. J. Environ. Chem. Eng..

[11] Baroulaki I., Karakasi O., Pappa G., Tarantili P., Economides D., Magoulas K. (2006). Preparation and study of plastic compounds containing polyolefins and post used newspaper fibers. Compos. Part A Appl. Sci. Manuf..

[12] Saleem J., Riaz M.A., McKay G. (2018). Oil sorbents from plastic wastes and polymers: A review. J. Hazard. Mater..

[13] Bazargan A., Hui C.W., McKay G. (2013). Porous Carbons from Plastic Waste. Adv. Polym. Sci. Springer.

[14] Rahimi A., García J.M. (2017). Chemical recycling of waste plastics for new materials production. Nat. Rev. Chem..

[15] Kahlen S., Braun H., Liu Y., Gahleitner M., Hubner G. (2020). Compatibilization of Recycled Polyethylene-Polypropylene Blends. U.S. Patent.

[16] Kulshreshtha B. (2018). Polypropylene-Polyethylene Composition with Improved Flowability. U.S. Patent.

[17] Kahlen S. (2021). Polypropylene Polyethylene Mixture Upgrading. U.S. Patent.

[18] Kahlen S., Jerabek M. (2021). Upgraded Recycled Polypropylene Rich Polyolefin Material. U.S. Patent.

[19] Kahlen S. (2022). Upgraded Recycled Polyethylene Polypropylene Blend. U.S. Patent.

[20] Kahlen S. (2021). Upgraded Recycled Relatively Polyethyleen Rich Polyolefin Materials. U.S. Patent.

[21] Kulshreshtha B. (2020). Recycled Polyethylene-Polypropylene Blend with Compatibilizer KR20210137576A. U.S. Patent.

[22] Saleem J., Moghal Z.K.B., McKay G. (2023). Up-cycling plastic waste into swellable super-sorbents. J. Hazard. Mater..

[23] Saleem J., Baig M.Z.K., Luyt A.S., Shakoor R.A., Hafeez A., Ahsan I., Pradhan S., Pasha M., McKay G. (2023). A facile energy-efficient approach to prepare super oil-sorbent thin films. Energy Rep..

[24] Haleem A., Chen J., Guo X.-X., Hou S.-C., Chen S.-Q., Siddiq M., He W.-D. (2022). Radiation-induced synthesis of hydrophobic cryogels with rapid and high absorption of organic solvents and oils. Microporous Mesoporous Mater..

[25] Haleem A., Pan J.-M., Shah A., Hussain H., He W.-D. (2023). A systematic review on new advancement and assessment of emerging polymeric cryogels for environmental sustainability and energy production. Sep. Purif. Technol..

[26] Yuan J., Liu X., Akbulut O., Hu J., Suib S.L., Kong J., Stellacci F. (2008). Superwetting nanowire membranes for selective absorption. Nat. Nanotechnol..

[27] Herkenberg W. (1995). Method for the Removal of Oil from Oil Spills. U.S. Patent.

[28] Wei Q.F., Mather R.R., Fotheringham A.F., Yang R.D. (2003). Evaluation of nonwoven polypropylene oil sorbents in marine oil-spill recovery. Mar. Pollut. Bull..

[29] Patel S.U., Chase G.G. (2014). Separation of water droplets from water-in-diesel dispersion using superhydrophobic polypropylene fibrous membranes. Sep. Purif. Technol..

[30] Saleem J., McKay G. (2016). Waste HDPE bottles for selective oil sorption. Asia-Pacific J. Chem. Eng..

[31] O’Dowd C.D., Jimenez J.L., Bahreini R., Flagan R.C., Seinfeld J.H., Hämeri K., Pirjola L., Kulmala M., Jennings S.G., Hoffmann T. (2002). Marine Aerosol Formation From Biogenic Iodine Emissions. Nature.

[32] Saleem J., Moghal Z.K.B., Luyt A.S., Shakoor R.A., McKay G. (2023). Free-Standing Porous and Nonporous Polyethylene Thin Films Using Spin Coating: An Alternate to the Extrusion–Stretching Process. ACS Appl. Polym. Mater..

[33] Saleem J., Baig M.Z.K., Luyt A.S., Shakoor R.A., Mansour S., McKay G. (2022). Reusable Macroporous Oil Sorbent Films from Plastic Wastes. Polymers.

[34] Ou J., Zhao G., Wang F., Li W., Lei S., Fang X., Siddiqui A.R., Xia Y., Amirfazli A. (2021). Durable Superhydrophobic Wood via One-Step Immersion in Composite Silane Solution. ACS Omega.

[35] Vasanthan N., Salem D.R. (2001). FTIR spectroscopic characterization of structural changes in polyamide-6 fibers during annealing and drawing. J. Polym. Sci. Part B: Polym. Phys..

[36] Song J., Liang J., Liu X., Krause W.E., Hinestroza J.P., Rojas O.J. (2009). Development and characterization of thin polymer films relevant to fiber processing. Thin Solid Films.

[37] Pawar A.A., Kim A., Kim H. (2021). Synthesis and performance evaluation of plastic waste aerogel as sustainable and reusable oil absorbent. Environ. Pollut..

[38] Atta A.M., Brostow W., Datashvili T., El-Ghazawy R.A., Lobland H.E.H., Hasan A.M., Perez J.M. (2013). Porous polyurethane foams based on recycled poly(ethylene terephthalate) for oil sorption. Polym. Int..

[39] Qian Q., Liu G., Lang D., Guo C., Wang L., Wu R. (2022). Recovery of unsaturated polyester resin into oligomer for preparation of oil-water separation aerogel. Mater. Today Sustain..

[40] Lang X.H., Zhu T.Y., Zou L., Prakashan K., Zhang Z.X. (2019). Fabrication and characterization of polypropylene aerogel material and aerogel coated hybrid materials fsor oil-water separation applications. Prog. Org. Coatings.

